# Interleukin-1 Beta—A Friend or Foe in Malignancies?

**DOI:** 10.3390/ijms19082155

**Published:** 2018-07-24

**Authors:** Rebekka Bent, Lorna Moll, Stephan Grabbe, Matthias Bros

**Affiliations:** Department of Dermatology, University Medical Center, 55131 Mainz, Germany; Rebekka.Bent@unimedizin-mainz.de (R.B.); Lorna.Moll@unimedizin-mainz.de (L.M.); Stephan.Grabbe@unimedizin-mainz.de (S.G.)

**Keywords:** interleukin-1β, promoter, inflammasome, tumor, tumor-associated macrophage, myeloid-derived suppressor cell

## Abstract

Interleukin-1 beta (IL-1β) is induced by inflammatory signals in a broad number of immune cell types. IL-1β (and IL-18) are the only cytokines which are processed by caspase-1 after inflammasome-mediated activation. This review aims to summarize current knowledge about parameters of regulation of IL-1β expression and its multi-facetted role in pathophysiological conditions. IL-1 signaling activates innate immune cells including antigen presenting cells, and drives polarization of CD4+ T cells towards T helper type (Th) 1 and Th17 cells. Therefore, IL-1β has been attributed a largely beneficial role in resolving acute inflammations, and by initiating adaptive anti-tumor responses. However, IL-1β generated in the course of chronic inflammation supports tumor development. Furthermore, IL-1β generated within the tumor microenvironment predominantly by tumor-infiltrating macrophages promotes tumor growth and metastasis via different mechanisms. These include the expression of IL-1 targets which promote neoangiogenesis and of soluble mediators in cancer-associated fibroblasts that evoke antiapoptotic signaling in tumor cells. Moreover, IL-1 promotes the propagation of myeloid-derived suppressor cells. Using genetic mouse models as well as agents for pharmacological inhibition of IL-1 signaling therapeutically applied for treatment of IL-1 associated autoimmune diseases indicate that IL-1β is a driver of tumor induction and development.

## 1. Introduction

Many aspects of the multi-facetted biological roles of Interleukin-1 alpha (IL-1α), and its functional homolog, but structurally different relative IL-1β were elucidated by the Dinarello lab [1]. IL-1α and IL-1β constitute the founding members of the IL-1 family which by now comprises eleven members [2]. Of note, all IL-1 family members are expressed as zymogens and most require proteasomal cleavage to yield biologically active mature forms. Of these, pro-IL-1β and pro-IL-18 are unique as they are cleaved by activated inflammasomes [3]. These multi-protein complexes are assembled in response to exogenous and endogenous danger signals and induce activation of caspases that generate bioactive IL-1β and IL-18. IL-1β is generated and released by numerous distinct immune and non-immune cell types rapidly in response to inflammatory signals [1]. In contrast, IL-1α is rather constitutively expressed in many non-immune cell types, but mainly remains in the cytosol or bound to the cell surface released in case of cell necrosis [4].

IL-1β acts as an amplifier of immune reactions [1]. In agreement, IL-1 exerts potent pyrogenic activity. For quite a long time, IL-1 has been widely acknowledged as required for efficient initiation of innate and shaping of adaptive immune responses to resolve acute inflammations [2]. However, the view of IL-1β as a beneficial immune regulator has been challenged by the finding that gain-of-function mutations in components of inflammasomes result in excessive IL-1β production that contribute to autoimmune [5] and cause autoinflammatory [1] diseases. In addition, in the case of chronic inflammation, sustained IL-1β may promote both tumor induction and later on also tumor propagation by different mechanisms [2]. Interestingly, we and others have shown that a number of anti-tumor therapeutics induces IL-1β production [6].

This review article aims to summarize our current knowledge on the different layers of regulation of IL-1β expression, and the multi-facetted biological roles of this important cytokine under pathophysiological conditions with a focus on tumor development.

## 2. Production of IL-1β Requires Two Distinct Signals

The different signaling pathways and transcription factors which elevate IL-1β gene expression in response to proinflammatory mediators, including IL-1β itself, have been thoroughly assessed. Besides transcriptional regulation, the half-life of generated IL-1β mRNA is regulated on posttranscriptional level by RNA-binding proteins. Derived pro-IL-1β protein requires proteolytic cleavage to acquire functional activity. This step is mainly facilitated by active caspase-1 as the effector component of stimulation-induced multi-protein inflammasomes. Altogether, these different layers of regulation allow to fine tune IL-1β production under different pathophysiological conditions as early recognized in human monocytic cells [7,8].

### 2.1. Gene Expression

The gene structure, expressional regulation, and function of IL-1β are evolutionarily well conserved [9]. In most studies the characteristics of regulatory elements of the human IL-1β gene promoter have been studied in human monocytic cells.

#### 2.1.1. Transcriptional Gene Regulation

**Figure 1:**
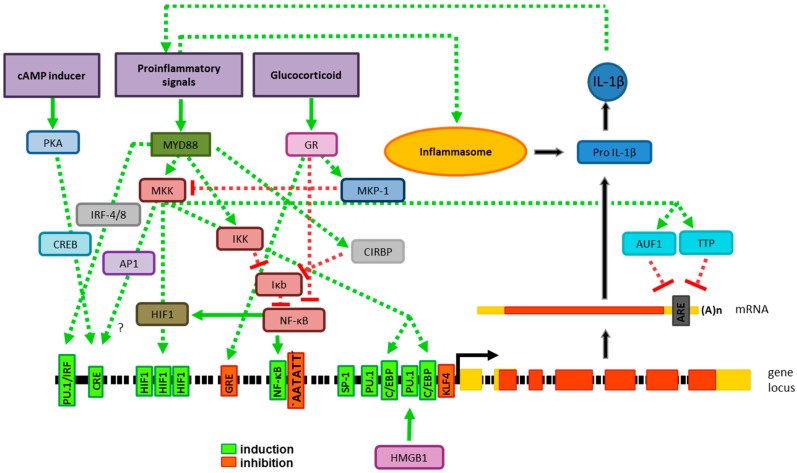
IL-1β expression. Transcriptional and posttranscriptional regulation results in expression of functionally inactive pro-IL-1β. Stimulatory (green) and inhibitory (red) activities exerted by stimuli, signaling adaptors and TF are indicated by arrows. TF binding sites (boxes) within the IL-1β gene promoter that induce (green) or inhibit (orange) gene expression are named. The transcription start site is drawn (black arrow). Exons (boxes) encompass non-coding (yellow) and protein-coding (orange) regions. The derived IL-1β mRNA, and the location of the AU-rich elements (ARE) engaged by RNA-binding proteins are depicted. Pro-IL-1β is cleaved by active inflammasomes which yields bioactive IL-1 β.

The TATA box containing the core promoter region of the IL-1β gene [10] harbors two bindings sites each for the myeloid-specific Ets-domain containing transcription factor (TF) PU.1 [11] and the leucine zipper containing TF CCAAT/enhancer binding protein (C/EBP-)β [12] (Figure 1). For keratinocytes, a specificity protein-1 (SP-1) binding site within the core promoter was identified as essential for steady state IL-1β mRNA expression [13]. Inflammatory stimuli such as toll-like receptor (TLR) ligands, tumor necrosis factor-α (TNF-α) or IL-1β itself [14] converge on activation of the signaling adaptor myeloid differentiation primary response 88 (MyD88) [15] which, besides other signaling pathways, is known to stimulate C/EBP [16]. In agreement, C/EBP-β was found to confer upregulation of IL-1β gene expression in response to the TLR4 ligand lipopolysaccharide (LPS) derived from gram-negative bacteria [17,18]. Of note, synergistic interaction of C/EBP-β with PU.1 did not require DNA binding of the former, but resulted from protein-tethered transactivation [12]. Furthermore, the endogenous danger molecule high mobility group box 1 protein (HMGB1) which is released by necrotic cells [19] was reported to enhance IL-1β gene expression via interaction with PU.1 as well [20]. Immediately upstream of the transcription start site, the IL-1β core promoter region also contains a functional binding site for the zinc finger-containing transcriptional repressor KLF4, which is induced by proinflammatory stimuli as well, and thereby limits IL-1β gene expression [21].

Upstream of the core promoter, a functional nuclear factor kappa-light-chain-enhancer of activated B-cells (NF-κB) binding site was reported to contribute to elevated IL-1β gene expression after stimulation [22]. MyD88 activation resulted in both direct MyD88/IκB kinase (IKK)-dependent degradation of IκB as a prerequisite for the release of NF-κB dimers and stimulation of mitogen activated protein kinase kinase (MKK) activity [23]. MyD88 activation was reported to stimulate cold-inducible RNA binding protein (CIRBP) which in turn down-regulated IκB expression and hence promoted IL-1β expression [24]. Lebedeva and Singh [25] identified a palindromic AT-rich sequence stretch located closely downstream of the NF-κB site to constitutively inhibit IL-1β promoter activity, but could not identify the transcriptional repressor involved. An inducible glucocorticoid response element (GRE) located upstream of the NF-κB site inhibited IL-1β gene expression in response to dexamethasone which activates the TF glucocorticoid repressor (GR) [26]. As another layer of negative IL-1β gene regulation, the activated GR was reported to directly inhibit LPS-induced NF-κB and activator protein-1 (AP-1) on protein level [27]. In addition, GR-induced MKP-1 was shown to repress LPS-induced IL-1β induction via MKK repression by direct protein-protein interaction [28].

Three closely spaced putative hypoxia-induced factor 1 (HIF1) binding sites were demonstrated to confer hypoxia-induced IL-1β upregulation [29]. Of note, HIF1 is also induced under stimulatory conditions in antigen presenting cells (APC) [30]. In line, the aforementioned HIF1 sites also contributed to IL-1β upregulation under according circumstances [29]. Furthermore, stimulation of myeloid cells with LPS-induced interferon response factors (IRF-)4 and IRF-8, and resulted in activation of PU.1 via protein kinase CK2 mediated phosphorylation [31]. IRF4/8 and PU.1 engaged a composite binding site and synergized to mediate IL-1β gene transcription [32]. Gray and coworkers [33] identified a cAMP-responsive element (CRE) adjacent to the composite PU.1/IRF binding site. This CRE site conferred stimulation of IL-1β gene expression in response to cAMP-elevating agents such as prostaglandin E2 in monocytes. In contrast, cAMP induction prevented LPS-mediated upregulation of IL-1β mRNA in astrocytes [34].

Different components of the extracellular matrix such as fibronectin, type I collagen, and laminin engage integrin receptors known to result in activation of protein kinase C (PKC) [35]. Activated PKC in turn stimulated AP-1 and thereby IL-1β gene expression [36]. In addition, PKC is associated with the MyD88 signaling cascade [37]. Thus far, no specific AP-1 binding site has been identified within the IL-1β gene promoter. Therefore, it remains possible that the CRE site identified by Gray and coworkers [33] may be engaged by TF of the AP-1 family as well. GATA-2 as a member of the “GATA” sequence binding TF family has also been implicated in MyD88/MKK transmitted upregulation of IL-1β mRNA expression [38]. However, none of the several potential GATA binding sites have been delineated as functionally active.

The overall accessibility of IL-1β gene promoter was found to be inhibited by DNA methylation in promyeloid cells while differentiated monocytes were devoid of methylated CpG motifs [39]. In models of LPS tolerance, phosphorylation of histone H3 serine around the IL-1β gene promoter was reduced and binding of the heterochromatin protein-1 (HP-1) was elevated, which correlated with repressed IL-1β gene expression [40].

In a systemic sequencing approach, Chen and coworkers [41] identified a number of single nucleotide polymorphisms (SNP) within the IL-1β gene locus, and performed functional assays identifying four SNP to affect overall promoter activity of stimulated monocytic cells. A mutation within the TATA box (SNP B15 [-31]: T ≥ C) diminished stimulation-induced promoter activity. At the same time, this mutation created a functionally active binding site for the TF Yin Yang 1 (YY1) [42], known to stimulate or silence gene expression [43]. SNP B14 (−511: C ≥ T) exerted a pronounced stimulatory effect on transcriptional activity of stimulated monocytic cells, whereas SNP B10 (−1464) and SNP B4 (−3737) moderately decreased overall promoter activity [41].

In a growing number of studies, the prevalence of those alleles within the IL-1β core (SNP B15 TT>CC) and proximal (SNP B14 TT>CC) promoter that conferred higher IL-1β gene expression was found to be associated with an enhanced susceptibility or aggravated course of disease. This included infectious diseases such as tuberculosis [44], neurodegenerative diseases such as Alzheimer’s disease [45], autoimmune and autoinflammatory diseases such as psoriasis [46] and diabetes type 2 [47], and the occurrence of tumors such as prostate cancer [48].

#### 2.1.2. Posttranscriptional Gene Regulation

Expression of human IL-1β has been demonstrated to be regulated not only on transcriptional level, but also by modulation of the IL-1β mRNA half-life. In this regard, Marucha and coworkers [49] demonstrated that treatment of human neutrophils with proinflammatory cytokines (IL-1β, TNF-α) transiently elevated both de novo IL-1β gene transcription as well as the half-life of IL-1β mRNA. On the contrary, IL-4 was identified as a negative regulator of IL-1β mRNA stability in LPS-stimulated monocytes [50]. In subsequent studies, the 3’-untranslated region of IL-1β mRNA was demonstrated to contain several functional AU-rich elements (ARE) which are engaged by a class of RNA-binding proteins [51]. Sirenko and coworkers [52] detected p38 mitogen-activated protein kinase (MAPK)-dependently activated ARE RNA-binding protein 1 (AUF1) to bind two of these ARE upon surface detachment of monocytes. AUF1 binding attenuated IL-1β mRNA stability. Similarly, we demonstrated that dendritic cells (DC) derived from mice deficient for the ARE-binding factor tristetraprolin were characterized by stronger upregulation of IL-1β mRNA in response to LPS treatment as compared with wild type DC due to a prolonged mRNA half-life [53].

### 2.2. Processing of Pro-IL-1β

The biogenesis of most IL-1 family members differs from other cytokines insofar as most IL-1 interleukins are generated as biologically inactive zymogen forms which are proteolytically cleaved [54]. In the case of IL-1β (and IL-18), N-terminal processing is facilitated in myeloid cell types by caspases which often serve as the effector component of inflammasomes. However, (partial) activation is also conferred by other proteases released by activated neutrophils and mast cells [55].

#### 2.2.1. Caspases

Cytoplasmic danger receptors that belong to the NLR protein family such as nucleotide-binding oligomerization domain-like receptor family leucine-rich repeat protein (NLRP) 1, NLRP3, and NLR family caspase recruitment domain containing 4 (NLRC4), or other types of danger receptors such as AIM2 (absent In melanoma 2), IFI-16 (interferon gamma inducible protein 16), RIG-1 (retinoic acid inducible gene-1), and MEFV (mediterranean fever) induce the formation of multi-protein inflammasomes in response to engagement by according ligands [3]. For this, binding of receptor-specific danger signals such as LPS, which specifically activates NLRP3, or flagellin, which is recognized by NLRC4, initiates inflammasome formation via inclusion of the adaptor protein ASC (activating signal cointegrator). These canonical inflammsomes recruit caspase-1 which in a proximity-dependent manner becomes autoproteolytically self-activated [56]. Dimeric active caspase-1 then mediates cleavage of pro-IL-1β and pro-IL-18.

By now, most studies have assessed the properties of NLRP3-based inflammasomes. In response to inflammation-induced MyD88 signaling, NLRP3 expression is upregulated as a prerequisite for formation of NLRP3 inflammasomes [57]. Of note, NLRP3 expression is negatively regulated on posttranscriptional level by the myeloid-specific micro-RNA (miR-)223 [58]. miR-223 has been attributed potent anti-inflammatory function in macrophages [59] as a consequence of inhibition of TF belonging to the NF-κB, C/EBP and STAT (signal transducer and activator of transcription) families [60].

On protein level, NLRP3 activity is mediated by deubiquitination as induced by TLR ligands [61]. NLRP3 oligomerization and activation requires interaction with NEK (NIMA-related kinase) after NLRP3-stimulation associated potassium efflux [62]. In a positive feedback loop, NLRP3 contributes to activation of NF-κB after microbial infection and application of particulate adjuvants [63]. However, sustained NLRP3 inflammasome activity is inhibited both by long-term LPS priming as well as inflammation-induced type I interferon (IFN) [64]. In both cases, nitric oxide synthase type 2 is induced which generates nitric oxide resulting in nitrosylation of NLRP3. Moreover, A20, which confers both ubiquitin ligase and deubiquitinase activities, was identified as a NLRP3-specific inhibitor [65]. Prostaglandin-induced cAMP serves as an endogenous modifier of NLRP3 inflammasome activation as well. Prostaglandin E receptor 4 (EP4) signaling inhibited inflammasome activity in a protein kinase A (PKA) and exchange protein directly activated by cAMP (EPAC) independent manner [66]. On the contrary, signaling via EP3 elevated LPS-induced inflammasome activity [67].

Non-canonical inflammasomes lack a specific endogenous danger receptor, and are comprised of caspase-4 and -5 in human [68] or caspase-11 in mouse [69]. These inflammasomes are directly activated by intracellular LPS to confer pro-IL-1β and-IL-18 processing. In both cases, inflammasome activation may also result in lytic cell death, termed pyroptosis, due to cleavage-dependent activation of the pore forming protein gasdermin D [70]. More recently, an alternative NLRP3-associated inflammasome pathway has been described in human monocytes which is also induced by LPS or the endogenous TLR4 ligand oxidized 1-palmitoyl-2-arachidonoyl-sn-glycero-3-phosphorylcholine (OxPAPC) [71]. In contrast to canonical inflammasomes, this pathway does not result in pyroptosis.

Caspase-8 is activated by CD95 and TNF receptor 1 signaling, and mediates cell apoptosis [72]. However, in a number of studies, caspase-8 was shown to replace caspase-1 as the effector molecule of NLR (NLRP3, NLRC4) and non-NLR (AIM2) inflammasomes [73,74]. In addition, CD95 engagement [75] and LPS-induced formation of Fas-associated via death domain (FADD)/caspase-8 containing ripoptosomes [76] resulted in processing of pro-IL-1β in an inflammasome-independent manner.

In a growing number of studies, gain-of-function mutations of inflammasome initiating cytoplasmic danger receptor proteins were demonstrated to mediate an increase in overall IL-1β levels [5]. Gain-of-function mutations of NLRP3 [77] and NLRC4 [78] have been associated with autoimmune diseases which overlap in their symptoms, termed cryopyrin-associated periodic syndromes (CAPS). Interestingly, in CAPS patients with mutated NLRP3, catalytically active extracellular NLRP3 inflammasomes were detected [79]. These extracellular complexes were also internalized by other macrophages, spreading excessive IL-1β maturation.

Besides gain-of-function mutations of inflammsome proteins, a functional NLRP1 promoter polymorphism resulting in elevated gene expression was associated with increased susceptibility towards rheumatoid arthritis [80] and generalized vitiligo [81].

#### 2.2.2. Other Proteases

Neutrophils [82] and mast cells [83] store many different proteases in their granules that, upon release, exert divergent anti-pathogenic effects. Of these, elastase and closely related cathepsin G as well as chymase were shown to cleave pro-IL-1β at the same site, some residues upstream of the caspase cleavage site [55]. Extracellular maturation of IL-1β may be important in case of myeloid cells that shed pro-IL-1β containing membrane microvesicles [84] that are lysed by ATP [85]. In addition, the membrane integrity of pro-IL-1β producing leukocytes is disturbed by prolonged exposure to antimicrobial peptides [86] and as a consequence of inflammasome activation itself (pyroptosis) resulting in release of pro-IL-1β. Besides extracellular cleavage of pro-IL-1β, it remains possible that cathepsin G endogenously generated by leukocytes [87,88] may promotes cleavage of pro-IL-1β too.

## 3. IL-1β Signaling

### 3.1. The IL-1 Receptor

The Interleukin-1 receptor (IL-1R) family comprises 10 members which share a highly similar structure. They consist of extracellular immunoglobulin domains as well as a cytoplasmic Toll/IL-1 receptor (TIR) domain, except for IL-1R2 [89]. Of these receptors, only IL-1R1 and IL-1R2 are able to bind IL-1α and IL-1β as well as the receptor antagonist IL-1Ra [90]. Since IL-1R2 lacks the TIR domain, it cannot induce signaling upon substrate binding [91,92]. IL-1R2 constitutes a “decoy receptor” due to its capability to bind IL-1 without initiating IL-1 signaling [93]. IL-1R1 and IL-1R2 are expressed by a plethora of cells including immune cells and others [90]. Especially T cells, polymorphonuclear leukocytes, DC, monocytes, macrophages, and B cells express IL-1R1 and IL-1R2 [90]. Furthermore, IL-1R1 is expressed by most epithelial cells [90]. In mice, IL-1R2 is mainly apparent in neutrophils [94,95]. IL-1R3, the accessory chain of the IL-1R members, is expressed by all IL-1 responsive cell types [90].

### 3.2. IL-1β Signaling Cascade

**Figure 2:**
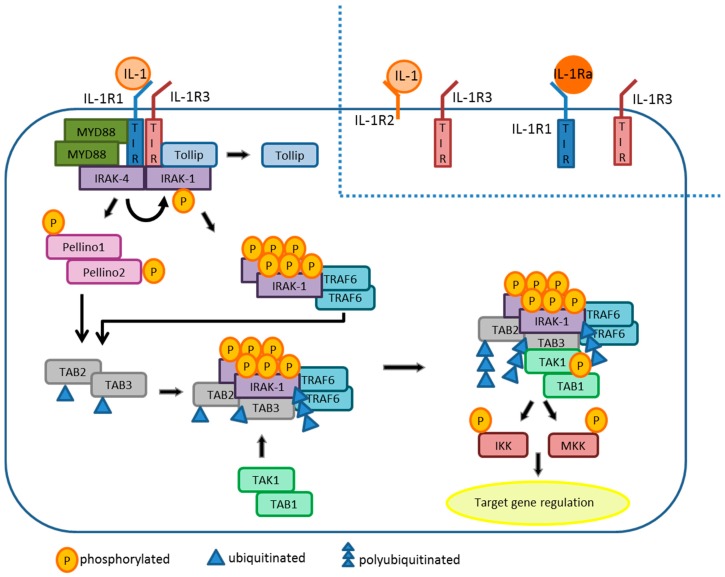
IL-1 signaling in target cells. Binding of IL-1 to IL1R1/3 results in cellular activation due to stimulation of IKK and MKK. IL-1 signaling is blunted in case of binding of IL-1 to the decoy receptor IL1R2/3, and by competitive binding of IL-1Ra to IL1R1/3.

Binding of the agonists IL-1α or IL-1β to IL-1R1 results in conformational changes of the receptor and in the formation of IL-1R1/IL-1R3 complexes (Figure 2) [96,97,98]. These conformational changes cause the TIR domain of IL-1R3 to get to close proximity to the TIR domain of IL-1R1 which induces IL-1 signaling [99]. Alternatively, IL-1 may engage IL-1R, and this complex subsequently engages IL-1R3, without inducing IL-1 signaling [90,100]. In addition, the receptor antagonist IL-1Ra can bind to IL-1R1, and thereby prevents the formation of the IL-1R complex [101].

Upon formation of the IL-1R complex, the two intracellular TIR domains of IL-1R1 and IL-1R3 recruit the TIR domain-containing adaptor protein MyD88 [102,103,104,105]. The attracted MyD88 proteins constitue oligomers which form a death domain (DD) important for subsequent signaling [106]. The DD of MyD88 recruits proteins of the IL-1R-associated kinase (IRAK) family. First, IRAK-4 binds to MyD88 followed by IRAK-1 and-2 [107,108], Toll-interacting protein (Tollip) [109] as well as Pellino-1 (PELI-1) and-2. PELI-1 and-2 are E3-ubiquitin ligases [110]. Non-phosphorylated IRAK-1 bound to Tollip is inactive [111]. When IRAK-1 binds to the IL-1R complex, it is phosphorylated and thereby activated through IRAK-4 activity and dissociates from Tollip and from the IL-1R complex [109,112]. IRAK-1 becomes hyper-autophosphorylated due to its activation by IRAK-4. Then, TRAF6 (TNF receptor-associated factor 6) is recruited to hyperphosphorylated IRAK-1 followed by the activation of its E3 ubiquitin ligase activity [113]. At the same time, IRAK-4 phosphorylates and activates PELI-1 and-2 [114,115]. TAK1 binding protein (TAB)2 and TAB3 are ubiquitinated by activated PELI or TRAF6 and recruit the TGFβ1-activated kinase (TAK)1/TAB1 complex [116]. This event results in a conformational change of the complex that leads to autophosphorylation and activation of TAK1 [117,118]. TAK1 phosphorylates and activates downstream kinases such as the MKK or IKK complex which regulate target gene expression [119]. IRAK-2 can replace IRAK-1, but it lacks the autophosphorylation activity of IRAK-1, and is therefore not targeted for fast degradation [120]. Thereby, IRAK-1 is a more stable signaling component of the IL-1 pathway [90].

### 3.3. IL-1Ra and IL-1R2

IL-1Ra is structurally similar to IL-1α and IL-1β. Therefore, it is a competitor for binding to IL-1R1 [121]. In contrast to IL-1α and IL-1β, IL-1Ra blocks the downstream IL-1 signaling pathway by preventing the binding of IL-1R3 to IL-1 binding IL-1R1 [98]. Interestingly, IL-1Ra does not bind to IL-1R2 [122]. Four isoforms of IL-1Ra are known, one secreted isoform and three intracellular isoforms [121]. Only the secreted isoform has been characterized yet [122]. Bellehumeur and coworkers showed that human endometrial cells respond to IL-1β by increasing the levels of IL-1R1 and IL-1Ra by gene transcription, which IL-1R2 showed an enhanced mRNA stability [123].

A condition termed deficiency of interleukin-1 antagonist (DIRA) in humans proves the importance of IL-1Ra for the regulation of IL-1β signaling. DIRA results in lethal sterile multi-organ inflammation if not treated with recombinant IL-1Ra [90,124]. Furthermore, a variable number of tandem repeats polymorphism in the IL-1Ra gene (IL-1RN) was shown to be associated with an increased risk to develop cancer [59]. Furthermore, a study showed that the synonymous coding variant rs315952 of IL-1RN is strongly correlated with improved survival after septic shock due to increased plasma levels of IL-1RA [125].

IL-1R2 exists as a membrane-bound and as a soluble form. Both can bind both IL-1α and IL-1β, with higher affinity to IL-1β, and inhibit the IL-1 signaling cascade by preventing both cytokines from binding to IL-1R1 [90,126,127]. The soluble form of IL-1R2 is either generated through proteolytic cleavage of the extracellular region [94,128,129,130,131] or by alternative splicing [101,132,133]. Both IL-1Ra and IL-1R2 regulate IL-1 signaling by different mechanisms, and thereby reduce IL-1-mediated inflammation [9].

IL-1R2 was shown to play important roles in several diseases such as diabetes, atherosclerosis, sepsis, Alzheimer’s disease, autoimmune inner ear disease (AIED), ulcerative colitis, and arthritis [134]. AIED patients do not express the full length and thereby functional IL-1R2 in their peripheral blood cells which suggests a protective function of IL-1R2 in AIED [135]. A recent study by Mora-Buch and coworkers showed that IL-1R2 production by epithelial cells was increased during remission of ulcerative colitis [136]. These results were confirmed by the finding that IL-1R2 transcription was reduced in patients that relapsed within 1 year compared to patients in remission. In addition, Shimizu and coworkers identified IL-R2 as an important regulator of arthritis. IL-1R2 inhibits collagen-induced arthritis by downregulation of the IL-1 signaling cascade in macrophages [95].

## 4. Role of IL-1 in Inflammation

IL-1 was early identified as an important hematopoietic factor that induced expression of colony stimulating factors on progenitor cells as well as leukocytes and stroma cells [137]. In line, administration of IL-1 to lethally irradiated mice resulted in recovery of the myeloid compartment [138].

IL-1 has been strongly implicated in the inflammatory response of immune cells due to its pronounced IKK [139] and MEK [140] stimulatory effects including upregulation of MyD88 [141]. In this regard, IL-1 constitutes a potent stimulator of APC as initially described by Koide and coworkers using recombinant IL-1α for murine splenic DC [142], and confirmed by Heufler and coworkers identifying IL-1β as the relevant factor for the maturation of Langerhans cells [143] that constitute the epidermal DC population. Furthermore, IL-1β was shown to promote the differentiation of monocytes to conventional DC [139] and to M1-like macrophages [144]. In addition, IL-1 was early demonstrated to support the proliferation of activated B cells and their differentiation to plasma cells [145]. IL-1 in combination with IL-2 promoted the expansion of natural killer (NK) cells as well as of CD4^+^ CD8^+^ T cells [146]. Moreover, IL-1β generated by activated APC induced type 1 immune responses. This was reflected by elevating frequencies of IFN-γ producing cytotoxic T lymphocytes (CTL) [147] and the polarization of αβ CD4^+^ T cells towards T helper cell type 1 (Th1) [148]. However, IL-1 was also reported to induce IL-4 receptor expression on CD4^+^ T cells [149] which is necessary for maintenance of Th2 cells. In combination with IL-6 [150] and IL-23 [151], IL-1β favored the differentiation of αβ CD4^+^ T cells towards Th17. Of note, IL-1β was described to counter-act TGF-β induced Foxp3 expression in CD4^+^ T cells, thereby inhibiting the differentiation of regulatory T cells (Treg) [152]. Moreover, IL-1β induced alternative splicing of Foxp3 in Treg resulting in a functional switch towards Th17 [153].

IL-1β not only promoted Th17 polarization via IL1R signaling, but also supported APC-induced Th17 production by CD4^+^ memory T cells. In this regard, human DC were reported to upregulate both surface-bound and soluble CD14 which interacted with T cell surface receptors promoting IL-17 production [154]. In the case of memory Th17 cells, IL-1β inhibited restimulation-induced IL-10 production [155]. Similar to αβ CD4^+^ T cells, IL-1 with IL-23 also induced IL-17 production by γδ CD4^+^ T cells [156].

IL-1 induced in the course of acute inflammation promoted the upregulation of adhesion receptors on immune and endothelia cells as a prerequisite for infiltration of leukocytes to sites of infection [157]. The key role of IL-1β in this regard was exemplified by Miller and coworkers demonstrating that neutrophil-mediated eradication of cutaneous *S. aureus* infection critically depended on IL-1 signaling [158].

Besides the beneficial role of IL-1β for clearance of infections, this cytokine has also been attributed to contribute to the severity of inflammatory diseases [124,159]. For example, elevated IL-1 signaling was demonstrated to cause neuronal cell death [160]. Of note, inflammasome activation and hence elevated IL-1β production has been observed in patients suffering from epilepsy [161], stroke [162], Alzheimer’s disease [163], and other neurological disorders [164]. IL-1 is also an important mediator of autoimmune diseases as evidenced by the therapeutic efficacy of IL-1Ra or IL-1β specific antibodies for treatment of rheumatoid arthritis [159] and gouty arthritis [165]. Autoreactive T cells are a central component of autoimmune diseases [166], and (myeloid) cells that generate IL-1 contribute to disease progression [167]. In contrast, so-called autoinflammatory diseases are largely caused by dysregulated IL-1β production [1]. As described above, hereditary gain-of-mutations in inflammasome components are causative for diseases such as CAPS (see Section 2.2.1) that are also treated by IL-1 signaling inhibition. Overproduction of IL-1β has also been observed in case of type 2 diabetes. Glucose itself induced NF-κB activity and hence IL-1β expression in β cells [168]. Furthermore, minimally oxidized low density lipoproteins were found to be elevated in diabetic patients which triggered IL-1β gene expression via TLR4 engagement [169]. In the same study, accumulation of islet amyloid polypeptide was shown to mediate NLRP3 inflammasome activation in islet macrophages [170]. In general, IL-1β mediated destruction of pancreatic β islet cells which was counteracted in patients by administration of IL-1Ra [171].

## 5. Role of IL-1β in Tumor Development

Proinflammatory innate cytokines including IL-1, TNF-α, and IL-6 are crucial to resolve acute inflammations. However, high levels of innate cytokines as apparent in chronic inflammation may promote tumor development by driving sustained NF-κB activation [172] and mitogen activated protein kinase (MAPK) activity [173]. Moreover, these cytokines promote expression of pro-tumorigenic genes that encode cell cycle, antiapoptotic and other proteins. Additional signaling pathways including protein kinase B (AKT) or Wingless (WNT) have been attributed tumor-inducing properties [174].

The development of multiple myeloma constitutes one example of a direct link between aberrant IL-1 production and tumor induction. Multiple myeloma is characterized by an accumulation of monoclonal plasma cells [175]. Smoldering multiple myeloma constitutes an early stage of multiple myeloma as patients develop the disease within one to two years later [176]. The driving mechanism is high level production of IL-1β by plasma cells which induces IL-6 in stromal cells [177]. IL-6 in turn promotes the development of malign plasma cells. Treatment of patients expected to develop multiple myeloma with IL-1Ra and the glucocorticoid dexamethasone proved successful to prevent further disease progression [178].

### 5.1. Anti-Tumorigenic Effects of IL-1β

In agreement with the importance of IL-1 for the induction of type 1 and type 17 antigen-specific T cell responses, the potential of recombinant IL-1 to induce pronounced anti-tumorigenic effects has early been assessed. For example, Nakamura and coworkers [179] demonstrated that intratumoral injection of IL-1a resulted in regression of different types of transplanted syngeneic tumors, including sarcoma, melanoma, and adenocarcinoma. North and colleagues [180] observed tumor regression only when IL-1 was applied intratumorally to tumors that had grown for a week, but not at earlier timer points. Due to the finding that T cell-depleted mice showed no signs of IL-1 induced tumor regression, the authors suggested that IL-1 was critical for the expansion of tumor antigen-specific T cells. Besides these therapeutic effects, Allen and coworkers [181] demonstrated a protective role of IL-1β in mouse models of chemically induced colitis and colon carcinoma. In myeloma-resistant T cell receptor (TCR)-transgenic severe combined immunodeficiency (SCID) mice [182] injected with myeloma cells, in vivo neutralization of IL-1 resulted in a decreased production of IFN-γ by tumor-specific Th1 cells and attenuated infiltration of macrophages and tumor growth [183].

While application of recombinant IL-1 exerted anti-tumor effects in a number of mouse studies (see above), in several clinical trials systemic application of IL-1 yielded only poor effects on hematopoiesis and tumor development, but significant toxicity [184]. To prevent widespread cytotoxicity, but to promote APC activation, IL-1 was encapsulated into microspheres [185]. These IL-1 loaded microspheres were preferentially internalized by macrophages [186]. Intratumoral application of such IL-1 delivery systems into fibrosarcoma-burdened mice delayed tumor growth which was associated with tumor cell necrosis and strong infiltration of leukocytes.

Tumor cell necrosis resulting from pronounced type 1 [187] and type 17 [188] immune responses and from chemotherapy [189] may further support the IL-1 signaling cascade by the release of mitochondrial ATP [190]. Extracellular ATP binds the purinergic receptor P2RX7 and thereby stimulates the NLRP3 inflammasome of myeloid APC [191]. Accordingly, mice deficient for NLRP3 inflammasome activation and IL-1 and IL-17 signaling [192] showed no response to chemotherapy with immunogenic cell death inducers such as anthracyclines. On cellular level, DC that lacked inflammasome activity when incubated with dying tumor cells that released ATP as a NLRP3 inflammasome inducer were no efficient CTL inducers [191]. Moreover, in anthracycline-treated breast cancer patients, an association between the occurrence of an allele of the P2RX7 receptor with reduced binding affinity to ATP and the beneficial effect of chemotherapy was observed. In contrast to these examples of anti-tumor effects of IL-1β, a pro-tumorigenic role of this cytokine has been demonstrated in numerous studies, which is outlined below.

### 5.2. Tumor-Promoting Effects of IL-1β

**Figure 3:**
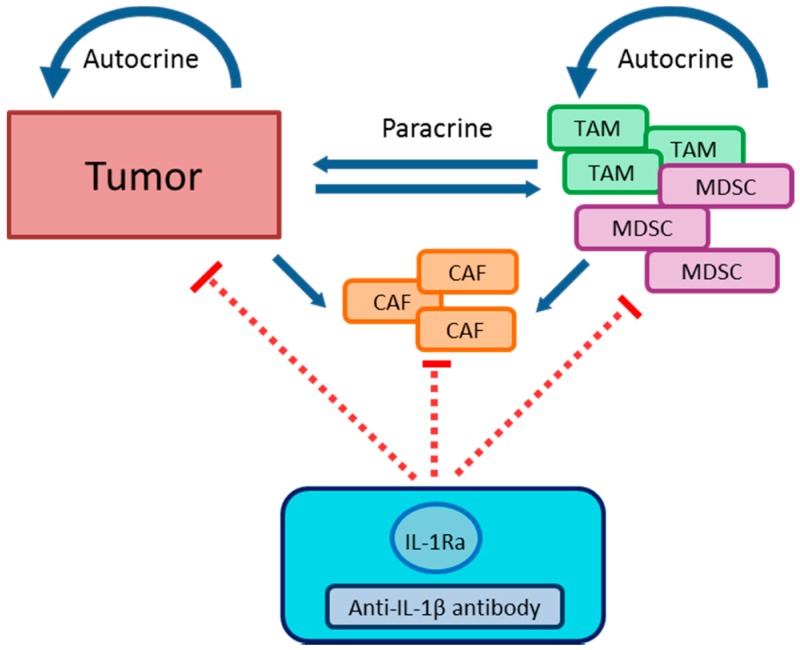
Effects of IL-1 in the tumor microenvironment. IL-1 is expressed by tumor cells, and predominantly by regulatory immune cell populations such as TAM and MDSC in the tumor microenvironment. The interleukin exerts pro-tumorigenic autocrine and paracrine effects on several levels in these cell types as well as in CAF which themselves do not generate IL-1 (blue arrows). Protumorigenic effects of elevated IL-1 on these different cell types are counter-acted by administration of IL-1Ra which competes with IL-1(α and β), and thereby prevents IL-1 signaling (red arrows). In numerous studies, TAM and MDSC were demonstrated to specifically produce IL-1β. In accordance, application of IL-1β specific antibodies intended to prevent induction of IL-1 signaling was shown to confer anti-tumorigenic effects as well.

IL-1 plays a key role in carcinogenesis and tumor growth [193], due to its importance as a key downstream mediator of inflammation [194]. Increased levels of IL-1β in body fluids are correlated in experimental tumor models and in cancer patients with bad prognosis, carcinogenesis and invasiveness of the tumor [195,196,197]. The key mechanisms by which IL-1β promotes tumor development are driving chronic non-resolved inflammation [193], endothelial cell activation [194], tumor angiogenesis [195] and induction of immunosuppressive cells (Figure 3). Altogether, these mechanisms account for suppression of the adaptive immunity, tumor promotion and metastasis [193].

#### 5.2.1. Tumor Development Caused by Chronic Inflammation

By now, chronic inflammation is recognized as one of the hallmarks in carcinogenesis, tumor progression, and metastasis [198,199]. Chronic inflammation results from the inability of the body to resolve an acute inflammatory response. Persistent inflammation can trigger the development of various tumors such as colorectal cancer, gastric cancer, mucosa-associated lymphoid tissue cancer, lung cancer, bladder cancer, and HCC [200,201,202]. Hepatic inflammation for example as induced by hepatitis C virus infection is a cytokine driven disease, especially via IL-1β, triggering the transition from non-alcoholic fatty liver disease to severe fibrogenesis, and finally hepatocellular carcinoma [203]. IL-1β is induced by a *Helicobacter pylori* infection, the most common chronic bacterial infection, and is the etiological agent for gastric cancer [204]. The association of gastritis and gastric tumor development has been shown to be strongly associated with genetic polymorphisms in the IL-1β locus [205]. IL-1β therefore enhances the number of gastric tumors [206]. Another significant mechanistic link between IL-1β and an increased risk of gastric cancer is the induction of aberrant DNA methylation via this interleukin [207]. IL-1β is also about 2–3-fold upregulated in small airway epithelial cells of chronic obstructive pulmonary disease (COPD) patients and seems to influence COPD airway inflammation [202]. Because COPD is a known risk factor for developing lung cancer [208], IL-1β seems to play a profound role in this regard as well. Additionally, the development and invasiveness of tumors induced by the chemical carcinogen 3-methylcholanthrene (3MCA) is promoted by IL-1β [209]. Furthermore, the induction of immunosuppressive myeloid-derived suppressor cells (MDSC), regulatory T cells (Treg), tumor-associated macrophages (TAM) [210] and NK cells [211] is a consequence of chronic inflammation. Altogether, IL-1β plays a major role already in the transition of chronic inflammation to tumor induction.

#### 5.2.2. Tumor Microenvironment

##### Immunosuppression

The tumor microenvironment is characterized by dominant immunosuppression. It is induced by myeloid regulatory cells such as neutrophils [212,213], TAM [214,215,216], MDSC [217,218,219,220], and regulatory DC [221,222]. In addition, Treg and carcinoma-associated fibroblasts (CAF) contribute to the immunosuppressive milieu [222,223,224,225]. In a head and neck squamous cell carcinoma (HNSCC) mouse model inflammasome activation and thereby IL-1β upregulation was shown to cause an insufficient anti-tumor reactivity via the immunosuppressive network of MDSC, TAM and Treg [226]. MDSC and Treg have also been illustrated to be key negative regulators of the immune system in melanoma patients [227,228,229].

##### TAM

Macrophages particularly populate the tumor microenvironment and are highly prevalent in inflammation-mediated tumors. These cells are called alternatively activated (M2) macrophages [174,230]. In general, macrophages that infiltrate tumor tissues are driven by tumor-and T cell-derived cytokines to acquire a polarized M2 phenotype characterized by production of IL-4, IL-13, IL-10, and glucocorticoid hormones [227,231]. These mediators influence all aspects of tumor growth and progression, providing a nurturing niche for cancer stem cells, and promoting angiogenesis, e.g., by IL-1β, dependently expressed vascular endothelial growth factor (VEGF), paving the way for metastasis, and taming adaptive immune response [193,216].

The sabotage of anti-tumor immunity is achieved by impairing T cell activation and by inhibiting M1 macrophage-mediated innate anti-tumor immune responses [227]. TAM provide an inflammatory microenvironment via inflammasome activation and IL-1β production which promotes, for example, breast cancer progression [232]. IL-1β induces CCL2 expression in TAM and tumor cells as well, regulating myeloid cell recruitment into tumor tissue [232,233].

##### MDSC

MDSC are a heterogeneous population of immature myeloid cells that are precursors of DC, macrophages, and granulocytes [234]. They are induced by tumor-derived soluble factors [235]. IL-1β is a tumor-associated factor leading to expansion and migration of MDSC, being regulated by the IL-1RI/NF-κB pathway [236]. MDSC accumulate in the blood, lymph nodes, bone marrow and at tumor sites in most patients and in tumor-burdened animals. These cells downregulate immune surveillance and antitumor immunity by multiple mechanisms, thereby facilitating tumor growth [227,234,237,238,239,240,241,242,243].

It could be shown that mice injected with 4T1 mammary carcinoma tumor cells transfected with an IL-1β expression vector propagated significantly higher levels of MDSC compared to mice carrying the same tumors but not secreting IL-1β [244]. Furthermore, the number of monocytic (mo-)MDSC was significantly increased in the prematastatic lungs of tumor-bearing mice, thus promoting tumor cell arrest on endothelial cells and metastasis [245]. The translational importance of IL-1β as a driver of MDSC propagation is underlined by the finding that in peripheral blood of advanced melanoma patients an increased level of serum IL-1β was associated with the frequency of mo-MDSC and Treg. An enrichment of circulating mo-MDSC significantly correlated with decreased progression-free survival of melanoma patients [246]. Furthermore, IL-1β upregulated cyclooxygenase-2 (COX-2), which encodes prostaglandins that mediate MDSC propagation [247]. MDSC produce IL-1β and other pro-inflammatory molecules activating tissue-resident endothelial cells to produce VEGF and other angiogenic factors [248]. These proangiogenic factors induced endothelial cell proliferation and blood vessel formation. Besides direct effects of IL-1β and products of its target genes on the expansion of the MDSC pool, IL-1β induced CC-chemokine ligand 2 (CCL2) in macrophages and tumor cells. Elevated CCL2 within the tumor microenvironment promoted the recruitment of C-C chemokine receptor type 2 expressing (CCR2^+^) myeloid cell types, including MDSC and TAM [232].

Furthermore, IL-1β promotes cancer cell adhesion and hepatic metastases by up-regulation of vascular cell adhesion molecule (VCAM-1) on hepatic sinusoidal endothelium [249,250]. It was also revealed, that IL-1β expression in lungs of melanoma-bearing mice is facilitated by lung-recruited mo-MDSC, resulting in enhanced E-selectin expression [245].

##### CAF

Another key player within the tumor microenvironment is the group of carcinoma-associated fibroblasts (CAF) which contribute to the growth, expansion, and dissemination of cancer cells [251]. CAF originate from the activation of resident fibroblasts or a wide spectrum of other precursor cells such as smooth muscle cells, bone marrow-derived mesenchymal stem cells, epithelial cells, carcinoma cells, and many more [252,253]. CAF activation is triggered by a variety of stimuli, including cancer cell-derived TGF-β1 (tumor growth factor-β1), PDGF (platelet-derived growth factor)α, PDGFβ, basic fibroblast growth factor (bFGF), and IL-6 [254]. CAF contribute to the malignant aggressiveness of tumors through the induction of pro-inflammatory factors including IL-1β, IL-6/8, CCL2, CXCL (C-X-C motif ligand) 1/2/3/12, COX-2, osteopontin (OPN), and intercellular adhesion molecule 1 (ICAM-1) [225,251]. IL-1β was also identified as a stromal-acting chemokine secreted by ovarian cancer cells, suppressing p53 protein expression in CAF. p53 is known to maintain genomic integrity through apoptosis regulation despite DNA damage or instability and also regulates inflammation [255]. Knockdown of p53 expression significantly enhanced the expression and secretion of chemokines IL-1β, IL-6, IL-8, growth regulated oncogene-alpha (GRO-α), and VEGF. In vivo, a significant increase in mouse xenograft ovarian cancer tumor was seen as well [256]. Thereby, epithelial cancer cells use IL-1β as a communication factor to generate a pro-tumorigenic inflammatory microenvironment [256]. Liver metastasis of colorectal cancer is promoted by recruiting circulating CAF to facilitate prophase tumor construction tumorigenicity via an elevated expression of structural maintenance of chromosomes 1A (SMC1A), a subunit of cohesin [257]. Furthermore, it was demonstrated that signaling mediated by the G protein estrogen receptor (GPER) cells, induced by estrogen, triggered IL-1β and IL-1R1 expression in CAF and in breast cancer cells. Thus, the ligand-activated GPER triggers the Epidermal Growth Factor Receptor (EGFR)/extracellular signal–regulated kinases(ERK)/PKC signaling transduction pathway generating a feed forward loop that couples IL-1β induction by CAF to IL-1R1 expression by cancer cells [258]. This resulted in an up-regulation of IL-1β/IL1R1 target genes such as PTGES (prostaglandin E synthase), COX2, RAGE (receptor for advanced glycation endproducts), and ABCG2 (ATP binding cassette subfamily G member 2). Thereby, regulatory interaction between CAF and the cancer cells induces migration and invasive features in breast cancer cells via alterations of fibroblastoid cytoarchitecture and F-actin reorganization [258].

#### 5.2.3. Angiogenesis

IL-1β promotes tumor development as well by influencing angiogenesis. Tumor growth requires the sprouting of new capillaries from preexisting blood vessel. Microscopic in situ lesions of a tumor may exist for years without having their own microcirculation. For tumor progression, an angiogenic switch permits rapid growth and invasion [259,260]. It was shown that microenvironmental IL-1β leads to in vivo angiogenesis and invasiveness of different tumor cells [260]. Confirming these results, IL-1β secreting tumors showed enhanced tumor angiogenesis as evidenced by highly increased secretion of VEGF by malignant cells and blood vessel density in tumors [222,261,262]. In the early angiogenic response during tumor development, cross talk between VEGF and IL-1β takes place. IL-1β and VEGF seem to induce each other and are both essential for blood vessel growth. Furthermore, IL-1β induced CCL2 expression in macrophages and tumor cells [232]. CCL2 has been shown to have multiple pro-tumorigenic functions such as mediating tumor growth and angiogenesis [233].

#### 5.2.4. Metastasis

IL-1β paves the way for tumor induction and growth as well as metastasis. Tumor metastasis is a complex process, including: (i) tumor cell dissemination from primary foci; (ii) their infiltration into the vascular system; (iii) transition through the circulation; (iv) capture in capillary beds; and (v) formation of metastatic nodules in distant target organs [263]. In the process of tumor metastasis, the construction of a pre-metastatic microenvironment is an essential step. The environment of the distant metastatic target organs are rebuilt prior to the arrival of the tumor cells from the primary foci [264]. It could be shown that the number of mo-MDSC is significantly increased in the lungs of melanoma-burdened mice before the arrival of tumor cells. These MDSC secreted IL-1β, resulting in the expression of E-selectin in endothelia cells, being attributed to increased distant metastasis in the lung [245]. Primary tumor cells enable a distant engraftment by secretion of different growth factors [265], resulting in chemokine and cytokine upregulation in the metastatic targeted organ. In primary and metastatic tumor tissue, elevated levels of IL-1β could be found [232]. Furthermore, an augmentation of lung metastasis from A375M tumor cells could be observed after (IV) injection of IL-1β [266]. Upregulated expression of proinflammatory chemokines and cytokines resulted in recruitment of bone marrow-derived hematopoietic cells [267]. Mo-MDSC are a significant component of these recruited cells [245].

In experimental studies, NLRP3 and thereby IL-1β expression levels were correlated with the tumor size and the metastatic status of the sentinel lymph node [268]. IL-1β levels were also shown to correlate with implantation and liver metastatic growth in a melanoma model [269,270]. Intravenous application of IL-1β enhanced the hepatic metastasizing ability of intrasplenically injected B16 melanoma cells [270]. IL-1β and TNFα induced IL-18 release upregulating the expression of VCAM-1 on hepatic sinusoidal endothelium (HSE) and thereby promoting cancer cell adhesion and liver metastases [249]. An IL-1β dependent upregulation of VCAM-1 by HSE, accompanied by elevated expression of the very late antigen-4 (VLA-4) could also be confirmed in another study. Hereby, adhesion of the melanoma cells to HSE cells take place leading to an increased metastasis [271].

## 6. Anti-Cancer Agents Induce IL-1β

Chemotherapy causes numerous undesirable symptoms affecting physical functioning and quality of life. An association between cancer treatment-related symptoms and inflammatory cytokines could be shown, triggered by increased production and synergistic action of IL-1β and TNFα [272,273]. IL-1β processing and secretion is induced by numerous chemotherapeutic drugs and has been shown to either elevate IL-1β gene expression or inflammasome activity in myeloid cells (summarized in Table 1).

**Figure 4:**
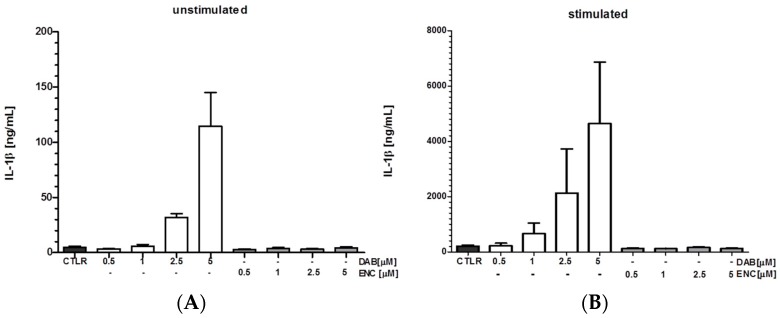
Effects of BRAF inhibitors on IL-1β production by DC. Murine bone marrow-derived DC at (**A**) unstimulated state or (**B**) treated with LPS [100 ng/mL] were (co)administered with varying doses of BRAF inhibitors. DAB, but not ENC induced IL-1β production in DC at either state of activation (mean ± SD; *n* = 4).

Most recently, we have identified the clinically used B-Raf proto-oncogene, serine/threonine kinase (BRAF)^V600E^ inhibitors (BRAFi) Dabrafenib (DAB) and Vemurafenib (VEM) to simultaneously mediate elevated IL-1β gene expression and inflammasome activation in DC and macrophages as an off target effect [6]. BRAFi are used for the treatment of metastatic melanoma in cases of a mutation in the signaling adaptor BRAF that results in ERK hyperactivation as an oncogenic target. This mutation (BRAF^V600E^) can be found in about 40% of all melanomas. We observed that DAB strongly upregulated IL-1β production in unstimulated and LPS-stimulated myeloid mouse APC and in unstimulated human primary DC. VEM also enhanced IL-1β secretion by mouse DC and macrophages, but not in human DC. Both BRAFi activated the NLRC4 inflammasome, and DAB additionally induced caspase-8 activation for pro-IL-1β processing. Contrarily, the newly developed BRAFi Encorafenib (ENC) had no effect on IL-1β production (Figure 4). The recently conducted phase III study for applying ENC plus the accordingly applied MEK1/2 inhibitor Binimetinib versus VEM or ENC administered alone showed improved progression-free survival and improved overall response of Encorafenib/Binimetinib treated patients. Treatment with ENC exerted less adverse events such as rash compared to VEM, although the frequency of grade 3 rash was slightly increased [274].

In our study on the IL-1β promoting effects of BRAFi, in the case of human primary DC isolated from peripheral blood, only a fraction of samples responded to DAB treatment with an induction of IL-1β [254]. These results are in line with the clinically observed side effect of DAB to cause fever in about 40–50% of patients treated with that BRAFi [275] which could be mediated by induced IL-1β due to its pronounced pyrogenic effects. Thus, these results suggest that genetic polymorphisms within the IL-1β or inflammasome genes determine the occurrence of these immunmodulatory off target effects of DAB.

The observation that chemotherapeutics may increase IL-1β levels in tumor patients may be detrimental for tumor therapy and therefore needs to be taken into consideration. Westborn et al. showed that treatment of SCID mice and Hmeso cells with the IL-1β inducing chemotherapeutic doxorubicin in combination with IL-1Ra resulted in a smaller tumor size compared to single cisplatin or IL-1Ra treatment [276]. Applying IL-1Ra in combination with cytotoxic chemotherapy seems such as a potential targeting option to reduce IL-1β dependent side effect of chemotherapeutic treatment. Furthermore, BRAFi treatment-induced tolerance in melanoma could be shown to result from a cytokine-signaling network involving TAM-derived IL-1β and CAFs derived C-X-C motif chemokine receptor 2 (CXCR2) ligands. The loss of host IL-1R signaling reduced melanoma growth in vivo as well [277]. These results could be confirmed in another study issuing the outcome of breast and lung carcinoma treatment with paclitaxel in combination with Anakinra. This combination treatment resulted in a reduced tumor burden. However, the combined therapy increased metastasis in mice lungs when compared to a monotherapy with paclitaxel. Thus, dual effects of blocking the IL1 pathway on tumor growth are possible. Therefore, treatments using “add-on” drugs such as the IL-1β blocker to conventional chemotherapy should be investigated in appropriate tumor models monitoring both the primary tumor and the induction of metastases [278].

## 7. Inhibition of IL-1 Signaling Inhibits Tumor Growth

### 7.1. Genetic Models

Chronic high-level expression of bioactive IL-1β is an important promotor of tumor development [209]. IL-1β is involved in the positive feedback loop of IL-1β/Akt/retinoic acid receptor α (RARα) signaling, and thereby transmits the oncogenic property of RARα in gastric carcinoma [279]. In transgenic mice the expression of human IL-1β induced spontaneous gastric cancer, correlating with early recruitment of MDSC to the stomach [236]. *H. pylori* infection was also shown to induce IL-1β production which played a crucial role in gastric inflammation. IL-1R1 deficient mice demonstrated attenuated infiltration of inflammatory cells, especially of macrophages and neutrophils, into the stomach of *H. pylori* infected animals compared with wild type (WT) mice [280]. Furthermore, reduced susceptibility to gastric carcinogenesis could be observed in IL-1R1^-/-^ mice. Similarly, IL-1β^-/-^ mice were characterized by a reduction of *H. pylori* induced gastric tumors [206].

IL-1β was reported to induce methylation-dependent gene silencing via nitric oxide (NO) production [280]. NO-induced aberrant DNA methylation is one of the major inactivating mechanisms of tumor suppressor genes. An absence of E-cadherin promotor methylation in IL-1R1^-/-^ mice was observed, therefore IL-1 induced methylation is likely to be one of the major mechanisms for chronic inflammation inducing cancer [206,280]. Therefore, gastric inflammation is considered as an inducer of DNA methylation and as a driving force in gastric carcinogenesis.

Additionally, IL-1β driven chronic Inflammation promoted the development and invasiveness of chemical carcinogen-induced tumors as well [196,260]. Mice deficient for IL-1β or IL-1R1 were protected against methylcholanthrene (MCA) carcinogenesis. At the site of carcinogen injection, a sparse leukocyte infiltration occurred in IL-1β deficient mice opposing the dense neutrophile infiltration in IL-1Ra^-/-^ mice. In WT and IL-1α^-/-^ mice leukocyte infiltration at this site was apparent being dominated by macrophages, whereas in the IL-1β^-/-^ mice cellular infiltration was nearly absent [209].

Another important aspect of tumor growth being modulated and influenced by IL-1β knockout is the development of metastasis. In a breast cancer model, mice deficient for caspase-1 and thereby having reduced IL-1β levels exhibited significantly lower numbers of lung metastasis and attenuated tumor growth [232]. In a human oral squamous cell carcinoma (OSCC) model NLRP3 expression levels correlated with tumor size, lymph node metastatic status and IL-1β expression. Down-regulation of NLRP3 expression using short hairpin RNA markedly reduced IL-1β production and OSCC cell proliferation, migration and invasion. Moreover, silencing NLRP3 expression significantly inhibited OSCC tumor growth in vivo [268].

### 7.2. Pharmacological Inhibition

As outlined above, inhibition of IL-1β signaling by using appropriate mouse models was shown to influence multiple key mechanisms of tumor growth such as chronic inflammation that triggered tumor development [193], accumulation of immunosuppressive cells in the tumor milieu [194], angiogenesis [195] within the tumor microenvironment, and metastasis [196]. Therefore, blockade of IL-1β may constitute an important therapeutic rationale to impair tumor development and progression. Moreover, it may serve to reduce therapeutic side effects and for palliative care [289].

Several types of agents are available to prevent the biological activtiy of IL-1β, including IL-1β specific antibodies (Canakinumab) [290] and IL1Ra (Anakinra) [290] as well as inflammasome inhibitors (Thalidomide, Parthenolide, and BAY-11-7082) [260,289,291]. The two IL-1 blocking agents Anakinra and Canakinumab have already been approved for the treatment of systemic inflammatory disorders and display an excellent safety profile [290].

### 7.3. Immuno-Suppression

In the tumor microenvironment in HNSCC, impairment of T cell responses resulted in an immunosuppressive state which is caused by MDSC, Treg and TAM [292,293]. TAM and MDSC can be recruited to the tumor side by IL-1β [232]. In agreement, in a mouse breast cancer model inhibition of NLRP3 effectively reduced the tumor-resident population of MDCS [288] and TAM [232]. Similarly, in a HNSCC mouse model, a reduction of MDSC, Treg and TAM frequencies was achieved by blocking NLRP3 inflammasome activation using the specific inhibitor MCC950 [226]. Comparable effects were observed in murine and human breast cancer models when applying Anakinra. In these studies, blockade of IL-1 signaling was accompanied by a lower myeloid cell accumulation in the tumor microenvironment whereas normal inflammasome activity contrarily lead to the infiltration of MDSC and TAM into the microenvironment [232]. Furthermore, it could be shown, using the mouse mammary tumor virus (MMTV)—inducible fibroblast growth factor receptor 1 (iFGFR1) transgenic mouse model, that inhibition of IL-1β activity in vivo resulted in reduced iFGFR1 induced mammary epithelial proliferation and attenuated formation of hyperplasic structures. Targeting IL-1β partially inhibited formation of early-stage mammary lesions in part through the induction of Cox2 [294].

### 7.4. Angiogenesis

Without appropriate vascularization tumor cells become necrotic and/or apoptotic. In several studies, angiogenesis was assessed by the recruitment of blood vessel networks into Matrigel plugs containing tumor cells that were inoculated s.c. in mice. In case of IL-1β^-/-^ mice, no vascularization of Matrigel plugs containing B16 melanoma cells occurred. Moreover, the incorporation of IL-1Ra to B16 containing plugs in WT mice inhibited the ingrowth of blood vessel networks into the plugs as well [260]. The necessity of IL-1β for angiogenesis and invasiveness of the tumor in vivo was also verified in DA/3 mammary and prostate cancer cell models [260].

VEGF and IL-1β were demonstrated to interact in an autocrine circuit using Matrigel plugs supplemented with B16 melanoma cells. IL-1β inhibition reduced tumor growth for an extended period. In contrast, specific VEGF neutralization initially resulted in an initial tumor inhibition, but followed by tumor recurrence [4]. Additionally, IL-1β was induced by leptin, leading to an increase in the expression of VEGF/VEGFR-2. Moreover, leptin upregulation of VEGF/VEGFR2 was partially mediated by IL-1 signaling [295]. This triad of cross talk among IL-1, VEGF, and leptin promoted angiogenesis in breast tumors. This interaction is an important driver of leptin-induced oncogenic action and a key link between obesity and cancer progression [296]. It could also be shown that NLRP3-dependent IL-1β promoted lymphangiogenesis by human macrophages via a S1P receptor 1(S1Pr1)-dependent pathway in vitro. The strong reduction of IL-1β in S1PR1 deficient TAM prevented lymphangiogenesis [297].

### 7.5. Metastasis

In a murine B16 melanoma metastasis model, intrasplenic injection of recombinant IL-1β or LPS increased experimental liver metastasis, while reduction of metastases and increased survival rates were observed following IL-1Ra treatment [269,270]. A similar outcome was confirmed in numerous studies in which exogenous IL-1Ra exerted an inhibitory effect of on lung and liver experimental metastasis of human and murine melanoma cells [249,250,291].

The results of the aforementioned studies led to the conclusion that inhibition of IL-1 signaling has a pronounced inhibitory effect on tumor growth by targeting different mechanisms of tumor development, such as: (i) reducing chronic inflammation which triggers tumor development; (ii) propagation of immune-suppressive cells; (iii) accumulation of regulatory cells in the tumor milieu; (iv) angiogenesis; and (v) metastasis.

## 8. Concluding Remarks

In the last decades, research has gathered a wealth of information on parameters that regulate IL-1β expression and on the multi-facetted role of IL-1β in pathophysiological conditions. IL-1β promotes APC-stimulatory activity and the upregulation of adhesion receptors on immune and endothelial cells as a prerequisite for the infiltration of leukocytes to sites of infection in acute inflammation. As a consequence of gain-of-function mutations of IL-1 expression regulating components, IL-1β is a driver of autoinflammatory diseases.

However, conflicting effects of IL-1β in terms of tumor development have been observed. Due to the induction of type 1 and type 17 antigen-specific T cell responses IL-1β exerts anti-tumorigenic effects. Contrarily, IL-1β within the tumor microenvironment can promote carcinogenesis, tumor growth, and metastasis by different key mechanisms such as driving chronic non-resolved inflammation, endothelial cell activation, tumor angiogenesis, and the induction of immune-suppressive cells. Clinically, a correlation between high levels of IL-1β and bad prognosis in cancer patients was observed. Therefore, numerous studies on IL-1β and its inhibition in animal and human tumor models were conducted. IL-1 signaling (Anakinra), and more specifically IL-1β (antibody-mediated) inhibition hereby seem to target key components of tumor development in most cases resulting in reduced tumor development.

Several drugs used for cancer treatment were shown to induce IL-1β production, which may lead to unwanted side effects. A combination of these drugs with IL-1Ra or an IL-1β blocking antibody reduced side effects and smaller tumor growth. Another study, however, showed that chemotherapeutic treatment with an additional IL-1β inhibitor also reduced tumor growth, but increased metastasis. Therefore, treatments using “add-on” drugs that inhibit IL-1 signaling in combination with conventional therapy should be investigated in appropriate tumor models looking at the primary tumor site as well as metastasis.

Overall, latest data suggest that (additional) IL-1β inhibition in numerous tumor models is a promising therapeutic option. Further consistent research in experimental mouse models is needed to assess the efficacy of IL-1β blockade in combination with chemotherapeutics or other drug treatment such as immunotherapy. Although growing evidence confirms beneficial effects of IL-1 signaling inhibition in tumor therapy, surprisingly, it is not standard treatment yet, even though the FDA approved IL-1 blockers such as Anakinra and Canakinumab for treatment of systemic inflammatory disorders and both drugs show an excellent safety profile.

To potentially enhance the overall efficacy of anti-tumor drugs known to induce IL-1β, the efficacy of additional IL-1 signaling blockade needs to be evaluated in tumor therapy.

## Figures and Tables

**Table 1:** Anti-tumor drugs that induce IL-1β.

Anti-Tumor Agent	Doses	IL-1β Enhancement Via			Effects		IL-1β Production			Incubation	Cell Type	Detection Method	Ref
		Gene Expression	Inflamma- Some	?	Synergistic	Additive	Amounts	Pro-IL-1β	IL-1β				
Dabrafenib	0.1; 1; 2.5; 5 μM	x	NLRC4	-	-	MTX	~10 ng/mL			24 h	hDC, mBMDM, splenic mye. cell pop	qPCR, FACS	[6]
Vemurafenib	0.1; 1; 2.5; 5 μM	-	NLRC5	-	-	-	~1 ng/mL			24 h	mBMDM, splenic DC	qPCR, FACS	[6]
Doxorubicin	2.5; 10; 25; 100 μM	-	NLRP3	-	-	-	max. 700 pg (10 μM)			8 h	mBMDM	ELISA	[281]
	5 μM	-	NLRP3	-	-	-	↑↑↑			18 h	mBMDM	WB	[273]
	5 μM	-	-	x	-	-	~45 fold		x	2, 12, 24 h	mBMDM, mouse blood	FACS, qPCR	[282]
	10 μM	-	NLRP3	-	-	-	6.5 pg/mL IL-1β			48 h	Hmeso/H2373 MM cells	ELISA/WB	[276]
	5 μM	-	NLRP3	-	-	-	~2300 pg/mL IL-1β		x	2, 2–18 h	mBMDC	ELISA/WB	[283]
Daunorubicin	0.1; 0.25; 1; 2.5 μM	-	NLRP3	-	-	-	230 pg/mL			8 h	mBMDM	ELISA	[281]
Melphalan	2.8 μM	-	NLRP3	-	-	-	↑↑↑			18 h	mBMDM	WB	[273]
Cisplatin	17 μM	-	NLRP3	-	-	-	↑↑			18 h	mBMDM	WB	[273]
	100 μM	-	NLRP3	-	-	-	2.5 pg/mL IL-1β			48 h	Hmeso/H2373MM cells	ELISA/WB	[276]
	50 μM	-	NLRP3	-	-	-	13.2 nmol/mg/min			?	proximal tubulus cells	ELISA	[284]
	20 mg/kgKG I.p	-		x	-	-	~13 pg/mg			72 h	mouse kidney tissue	ELISA	[285]
Vincrisitin	0.4 μM	-	NLRP3	-	-	-	↑↑			18 h	mBMDM	WB	[273]
Etoposide	33 μM	-	NLRP3	-	-	-	↑↑			18 h	mBMDM	WB	[273]
Paclitaxel	1 μM	-	NLRP3	-	-	-	↑			18 h	mBMDM	WB	[273]
	200 nM/L 25/50 mg/kgKG	-		x	-	-	~0.35 pg/mL		x	24 h, 6, 12, 24, 48 h	blood	ELISA, RT-PCR	[278]
	2 mg/kg	-	NLRP3	-	-	-	~220%.		x	1/2/3 weeks	macrophages	RT-PCR	[286]
	4 mg/kg	-		x	-	-	~1.7 fold		x	36 days	dorsal root ganglia	RT-PCR	[287]
Methotrexate	2.3 μM	-	NLRP3	-	-	-	↑			18 h	mBMDM	WB	[273]
Cytarabine	2 μM	-	NLRP3	-	-	-	↑			18 h	mBMDM	WB	[273]
5-Flu	1 μM	-	NLRP3	-	-	-	~60 pg/mL			24, 48, 72 h	mMDSC	ELISA	[288]
Gemcitabine	27 nM	-	NLRP3	-	-	-	~70 pg/mL			24, 48, 72 h	mMDSC	ELISA	[288]
Doxo. & Vincr	5 μM & 0.4 μM	-	NLRP3	-	+	-	↑↑↑			18 h	mBMDM	WB	[273]
Cispl. & Vincr	17 μM & 0.4 μM	-	NLRP3	-	-	+	↑↑			18 h	mBMDM	WB	[273]

WB, Western Blot; h, human; m, mouse; BMDM, bone morrow-derived macrophage; myel., myeloid; ?, unknown; ↑/↑↑/↑↑↑, light/ medium/high upregulation of IL-1β.

## Abbreviations

APC	Antigen presenting cell
BMDC	Bone morrow-derived macrophages
BRAFi	BRAF Inhibitor
CAF	Carcinoma-associated fibroblasts
DAB	Dabrafenib
DC	Dentritic cell
ENC	Encorafenib
HNSCC	Head and neck squamous cell carcinoma
IL-1α	Interleukin-1 alpha
IL-1β	Interleukin-1 beta
IL-1Ra	Interleukin-1 receptor
LPS	Lipopolysaccharide
MCA	Methylcholanthrene
MyD88	Myeloid differentiation primary response 88
MDSC	Myeloid-derived suppressor cells
NK	Natural killer cells
NF-κB	Nuclear factor kappa-light-chain-enhancer of activated B-cells
TAM	Tumor-associated macrophages
Th	T helper type T
TLR	Toll-like receptor
Treg	Regulatory T cells
VEGF	Vascular Endothelial Growth Factor
